# Flexible Ureteroscopic Guided Laparoscopic Ureteroplasty For The Treatment Of Ureteral Stricture

**DOI:** 10.1590/S1677-5538.IBJU.2024.0250

**Published:** 2024-05-20

**Authors:** Agustín Cabrera Santa Cruz, Alexandre Danilovic, Fabio C. Vicentini, Giovanni S. Marchini, Carlos Batagello, Fabio Torricelli, William C. Nahas, Eduardo Mazzucchi

**Affiliations:** 1 Serviço de Urologia Hospital das Clínicas Universidade de São Paulo São Paulo SP Brasil Serviço de Urologia, Hospital das Clínicas Universidade de São Paulo - USP, São Paulo, SP, Brasil;; 2 Hospital Alemão Oswaldo Cruz São Paulo SP Brasil Hospital Alemão Oswaldo Cruz, São Paulo, SP, Brasil;; 3 Divisão de Urologia Faculdade de Medicina Universidade de São Paulo São Paulo SP Brasil Divisão de Urologia, Faculdade de Medicina da Universidade de São Paulo - São Paulo, SP, Brasil

## Abstract

**Introduction:**

Ureteral stricture is often a consequence of urolithiasis or previous endourological procedures (1-3). Precisely delineating the stricture zone intraoperatively is crucial to minimize ureter shortening and target only the affected tissue (4, 5). Flexible ureteroscopy offers a significant advantage in this regard.

**Objective:**

This video aims to demonstrate the step-by-step technique of flexible ureteroscopic guided laparoscopic ureteroplasty for treating ureteral stricture caused by urolithiasis and prior endourological interventions.

**Patient and Methods:**

We present a case of a 36-year-old male with a history of urolithiasis and unsuccessful endourological treatments, including endoureterotomy and balloon dilation, diagnosed with re-stenosis of the proximal ureter of 1 cm through ureteroscopy and pyelography. He underwent a successful laparoscopic ureteroplasty. While the lead surgeon performed the laparoscopy, an assistant conducted the flexible ureteroscopy. Intraoperatively, using transillumination facilitated by the flexible ureteroscope, we can precisely identify the narrowed area, allowing for resection of only the damaged segment. Subsequently, we perform the end-to-end ureteroplasty, confirming its patency through the seamless passage of the ureteroscope. Upon completion, we employ a fat patch to safeguard the anastomosis.

**Results:**

The patient was discharged on the third postoperative day. Double J stent was removed six weeks after surgery. Symptoms resolved. Renal function improved: eGFR 49 to 67 ml/min. Furthermore, improvement was observed in the DTPA scan, and a decrease in hydronephrosis was noted on the follow-up tomography.

**Conclusion:**

Flexible ureteroscopy effectively identifies the stricture zone in laparoscopic ureteroplasty, enhancing surgical precision and outcomes. This approach is safe, effective, and reproducible, offering a valuable technique in the surgical treatment of ureteral strictures.

Available at: http://www.intbrazjurol.com.br/video-section/20240250_Danilovic_et_al
